# Authors’ Response to the Validity of Cortisol and Galvanic Skin Responses for Measuring Student Stress During Training

**DOI:** 10.2196/50902

**Published:** 2023-08-18

**Authors:** Shannon Toohey, Alisa Wray, John Hunter, Soheil Saadat, Megan Boysen-Osborn, Jonathan Smart, Warren Wiechmann, Sarah D Pressman

**Affiliations:** 1 Department of Emergency Medicine University of California, Irvine Orange, CA United States; 2 Department of Psychology Crean College of Health and Behavioral Sciences Chapman University Orange, CA United States; 3 Department of Psychological Science University of California, Irvine Irvine, CA United States

**Keywords:** augmented reality, AR, salivary cortisol, galvanic skin conductance, medical simulation, medical education

We appreciate the interest in our article [1] and the critiques provided [2]. We addressed many of these in our protocol but did not mention them in the manuscript due to word count limitations.

Regarding interperson variability of cortisol levels, participants completed a baseline survey including information about menstrual cycle; birth control use; cardiac disease; kidney disease; Raynaud disease; current medications; sleep behavior; exercise; and alcohol, tobacco, caffeine, and drug use. We collected information on pre-existing psychological traits including perceived stress, depression, posttrumatic stress disorder, emotion, and stressful life events. Other short-term covariants were addressed the morning of the simulation with questions about sleep, wake-up time, caffeine consumption, and food consumption. For the analysis, we used a saturated multivariable model and backward-eliminated variables that failed to attain statistical significance. We found no significant impact of these factors on cortisol levels. Furthermore, the long-term covariants were addressed by the within-subjects study design.

Regarding timing of cortisol measurement, the first cortisol sample was just over 20 minutes after the start of the simulation. We started the 15-minute timer for the acute stress sample halfway through the standard 10-minute simulation. Psychological stress research has long established that responses to acute stress typically rise in 15-20 minutes. A change can be seen as soon as 10 minutes post acute stress [3], while 15 to 25 minutes is the research standard to see the maximum rise, and stress responses are already decreasing 30 minutes poststressor induction [4].

While we considered alternative physiologic markers such as α-amylase, we decided to use galvanic skin response (GSR) instead. We felt that α-amylase would be a less specific measure of sympathetic nervous system (SNS) activity compared to GSR. While α-amylase can be used as a general autonomic nervous system marker of stress, even the researchers who originally postulated it as an SNS measure question its validity these days [5].

Regarding products on hands impacting GSR data, we told participants to wash their hands with water only if they had products on their hands. While we did not measure the number of participants who did so, the vast majority did due to their habit of using hand sanitizer on arrival to the simulation center. While excessively sweaty palms would impact the GSR data, our hope was that the concurrent measurement of cortisol levels would provide an alternate measure for these participants.

We collected demographic information including sex, age, ethnicity, BMI, and marital status, and found no statistically significant differences. We did not include socioeconomic information, as it is difficult to ascertain among full-time medical students who predominantly live off loans/grants. We agree that these factors and socioeconomic status could impact stress response, and these would be essential covariants in future studies.

There are many opportunities for future research, and including learning outcomes would be an important addition. Focusing on evaluating short-term and long-term learning outcomes, and comparing those outcomes to both the students’ perception of stress and the measured stress responses would help better understand the impact of stress on learning.

## Abbreviations

GSR: galvanic skin response
SNS: sympathetic nervous system
